# Microplastics Prevalence in Different Cetaceans Stranded along the Western Taiwan Strait

**DOI:** 10.3390/ani14040641

**Published:** 2024-02-17

**Authors:** Reyilamu Aierken, Yuke Zhang, Qianhui Zeng, Liming Yong, Jincheng Qu, Haoran Tong, Xianyan Wang, Liyuan Zhao

**Affiliations:** 1 Third Institute of Oceanography, Ministry of Natural Resources, Xiamen 361005, China; rylm@foxmail.com (R.A.); zhangyuke@tio.org.cn (Y.Z.); zengqianhui@tio.org.cn (Q.Z.); 13696742606@163.com (L.Y.); qujincheng@tio.org.cn (J.Q.); 2 Key Laboratory of Marine Ecological Conservation and Restoration, Ministry of Natural Resources, Xiamen 361005, China; 3 Fujian Provincial Key Laboratory of Marine Ecological Conservation and Restoration, Xiamen 361005, China; 4 Museum of Biology, Xiamen University, Xiamen 361005, China; simontong424@gmail.com

**Keywords:** microplastics, cetacean, intestine, Taiwan Strait, East China Sea, apex predators

## Abstract

**Simple Summary:**

Monitoring microplastics (MPs) in cetaceans is challenging due to difficulties in obtaining samples, especially for endangered species. In this study, we characterized MPs ingested in nine individuals of four cetacean species stranded along the western coast of the Taiwan Strait including not only small coastal dolphins but also large pelagic and deep-diving cetaceans. A total intestine length of 123.2 m was analyzed, and the color, shape, size, and polymer types of MPs were then identified. The mean abundance of MPs was 86.44 ± 12.22 items individual^−1^, which was slightly higher compared with the same species from other waters worldwide. Moreover, a strong significant relationship between MPs abundance and intestine contents mass was identified. Transparent, fibrous MPs made from PET and PP were found to be predominant in the present study, indicating that the primary MPs source is from municipal and industrial sewage from clothing production and industry as well as from fisheries and aquaculture. Our study provides more information about the MPs pollution of endangered species in the western Taiwan Strait. It highlights the further risk assessment of MPs consumption in these threatened species.

**Abstract:**

Microplastics (MPs) pollution is of global concern, which poses serious threats to various marine organisms, including many threatened apex predators. In this study, MPs were investigated from nine cetaceans of four different species, comprising one common dolphin (*Delphinus delphis*), two pygmy sperm whales (*Kogia breviceps*), one ginkgo-toothed beaked whale (*Mesoplodon ginkgodens*), and five Indo-Pacific humpback dolphins (*Sousa chinensis*) stranded along the western coast of the Taiwan Strait from the East China Sea based on Fourier transform infrared (FTIR) spectroscopy analysis. Mean abundances of 778 identified MPs items were 86.44 ± 12.22 items individual^−1^ and 0.43 ± 0.19 items g^−1^ wet weight of intestine contents, which were found predominantly to be transparent, fiber-shaped polyethylene terephthalate (PET) items usually between 0.5 and 5 mm. The abundance of MPs was found at a slightly higher level and significantly correlated with intestine contents mass (*p* = 0.0004*). The MPs source was mainly likely from synthetic fibers-laden sewage discharged from intense textile industries. Our report represents the first study of MPs in pelagic and deep-diving cetaceans in China, which not only adds baseline data on MPs for cetaceans in Asian waters but also highlights the further risk assessment of MPs consumption in these threatened species.

## 1. Introduction

Microplastics (MPs) are considered as globally ubiquitous emerging pollutants and have especially entered into most marine environments, where they occur from coastal [1] to pelagic waters [2], from the surface sea [3] to the deep-sea [4], and also in marine sediments [5]. MPs not only come from biological degradation, photo-oxidative degradation, physical fragmentation, or chemical deposition of large plastic materials [6,7] but also include microbeads released from personal care products, cosmetics, pharmaceuticals, and synthetic fibers released from the production of garment textiles [8,9]. They enter the marine environment mainly from human activities, especially from aquaculture, fisheries, industry, and domestic sewage systems in coastal areas [6,10]. 

MPs are of considerable concern because they are potentially hazardous and widely distributed in many marine species, from low-trophic level zooplankton, fish, shrimp, and bivalves to apex predators such as cetaceans [11,12,13,14,15,16]. MPs can be directly absorbed from seawater or consumed and affect species by blocking digestive tracts, altering energy distribution, reducing growth rate, and causing pathological stress [17,18]. As apex mammal predators in the marine environment, cetaceans are appropriate sentinel species, who can provide early warning signs of existing or emerging health risks [19,20]. To date, MPs have been reported from the gastrointestinal tract of both filter-feeding baleen whales and teuthophagous and piscivorous toothed whales [10,15,21,22,23,24,25,26,27,28,29,30]. Although MPs do not represent a threat to cetaceans via entanglement or blocking like large plastic fragments do, they can act as carriers of toxic substances, such as heavy metals and persistent organic pollutants, which, combined with biomagnification and bioaccumulation, negatively affect health [31].

The western Taiwan Strait is affected by the Kuroshio current, Min-Zhe coastal current [32,33], and many upwellings in the Taiwan Strait [34], which bring nutrient-rich deeper water to the surface supporting five major fishing grounds with abundant fishery resources [35]. The frequent fishing activities and the increase in the use of plastic fishing gear have introduced MPs into the marine environment [36]. Meanwhile, coastal textile, shoe, and garment industries along the coast here might release MP-rich wastewaters into the marine environment [37,38]. More than 20 cetacean species were reported in this region, representing more than half of all cetacean species reported from China’s coastal waters [39]. 

Historically, MPs have been reported from the digestive tract of small coastal dolphins in Indo-Pacific humpback dolphins [26,40] and finless porpoises [24] from China, but without local studies of MPs in large pelagic and deep-diving cetaceans. We herein report on MPs from the intestine contents of cetaceans not only including coastal species but also pelagic and deep-diving species stranded along the western coast of the Taiwan Strait. The main goals of the present research are as follows: (1) to identify and compare the presence, frequency, and characteristics of MPs (including shape, color, polymer type, and size) in different cetacean species; and (2) to discuss the abundance level related factors and potential sources of MPs for these cetaceans. This investigation adds baseline data of MPs contamination status in cetaceans and their living marine environments in Asian waters. 

## 2. Materials and Methods

### 2.1. Sample Collection

**Figure 1:**
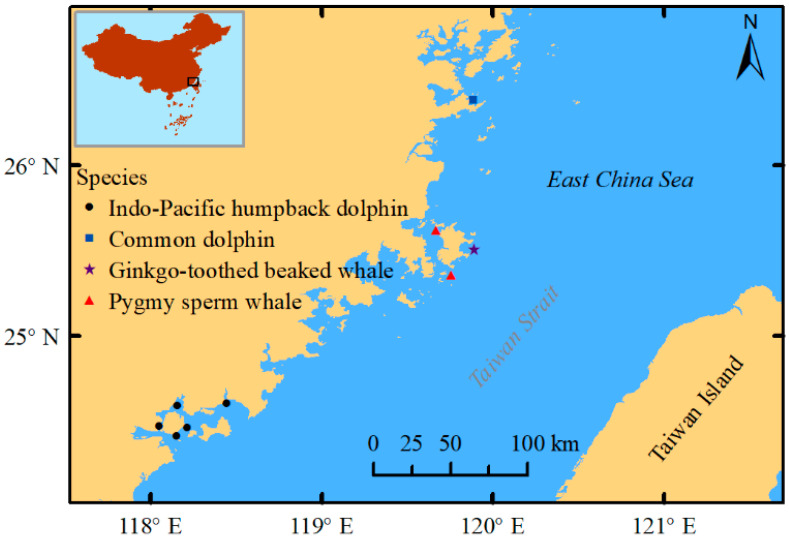
Cetacean carcass recovery sites along the western coast of the Taiwan Strait.

Nine stranded cetaceans including one common dolphin (*Delphinus delphis*), two pygmy sperm whales (*Kogia breviceps*), one ginkgo-toothed beaked whale (*Mesoplodon ginkgodens*) and five Indo-Pacific humpback dolphins (*Sousa chinensis*) were recovered mostly from 2019 to 2021, except for one in 2016. The common dolphin was recovered from Lianjiang, the pygmy sperm whales and ginkgo-toothed beaked whales were from Pingtan Island, and all five Indo-Pacific humpback dolphins were from Xiamen Bay off the western coast of Taiwan Strait from East China Sea. The recovery locations are shown in Figure 1. The carcass status ranged from freshly dead (code 2) to moderately decomposed (code 3) [41]. Carcasses were transported to the laboratory and stored at −20 °C. After later defrosting, prior to necropsy, external morphology was measured. Sex was determined by examination of reproductive organs. Sample management and dissection were performed in compliance with the introductory guide for the anatomy of marine mammalian necropsy [42]. For MPs analysis, the complete intestines were removed from each of the cetaceans, except for a 2.4 m subsample of intestine that was excised from the ginkgo-toothed beaked whale as some gut contents were used for other purposes (the 2 ends of the intestines were ligatured with cotton twine). To prevent contamination, the unopened intestines were subsequently double wrapped in aluminum foil and placed into separate sealed bags and stored at −20 °C. Necropsy and sampling were approved by the local fishery administration, with procedures conducted in accordance with all ethical codes and legal requirements in China.

### 2.2. MPs Isolation

According to the previous protocol [15] with some modifications, we isolated MPs from intestine contents in 19.5 m intestinal parts of one common dolphin (pelagic species); 48.7 m intestinal parts of two pygmy sperm whales and 2.4 m intestinal parts of one ginkgo-toothed beaked whale (deep-diving species); and 52.6 m intestinal parts of five Indo-Pacific humpback dolphins (coastal species). The weights and lengths of each intestine were measured after thawing. After rinsing the outer intestinal surfaces with filtered Milli-Q water repeatedly, the intestines were divided into several shorter segments to facilitate sample processing and collection. Each segment was dissected open, and the contents were collected and washed with filtered Milli-Q water through four nested sieves (0.1, 0.5, 1, 5 mm). Sieve residues were collected and rinsed into the clean glass containers using flowing filtered Milli-Q water. Empty intestines were re-weighed, with the difference in weight before and after their rinsing representing the wet weight of contents. The filtered 10% KOH solution was added into the glass containers to digest organic matter. Mixtures were then incubated at 60 °C at 120 rpm for 30–48 h until all biological material had completely dissolved. The digestion solution was transferred to the flotation device. 

The NaI solution (1.8 g cm^−3^) was added with an equal volume for density separation [43], and the funnel was covered with aluminum foil to prevent MPs pollution from the air. The latex tube was squeezed several times in succession to make the solution evenly mixed and then standing still. After 12 h flotation, the supernatant of dissolved mixture contents was then filtered through a GF/A Whatman filter (diameter = 47 mm, pore size = 1.6 μm) under a vacuum filter pump (Jin Teng GM-0.33A), which was then placed into a clean glass Petri dish with a lid and dried at room temperature. 

The process of identification and analysis of MPs was the same as our published paper [44]. All suspected MPs on filters were observed under a stereomicroscope (Leica M205 C) and photographed for color, shape identification, and measured size using NIS element imaging software (version 5.20.00, Nikon Corporation, Tokyo, Japan). All suspected MPs were transferred by tweezer to a clean aluminum-coated microscope slide (Thermo Fisher Scientific Waltham, MA, USA) and identified for polymer type by the Fourier transform infrared microscope (Micro-FTIR, Nicolet iN10, Thermo Fisher Scientific, Waltham, MA, USA) with a spectrum range set at 4000–400 cm^−1^, a collection time of 3 s, and with 16 scans made for each measurement under reflection mode. By comparing the standard spectra of polymers with the Bruker FTIR spectrum database using OPUS (Version 7.5), the composition of the microplastic polymer was accepted if the spectra matching degree was ≥ 70% [45].

### 2.3. Contamination Controls and Procedural Blanks

To reduce possible contamination, all work-bench surfaces were cleaned, and intestines were washed in a fume hood. The glassware was baked overnight (at 400 °C). All the tools and apparatus were pre-washed with filtered Milli-Q water three times. All containers were covered with aluminum foil when not in use. All experimental reagents were filtered through a 1.6 μm membrane filter prior to use. Any operator was required to wear a 100% cotton laboratory coat and blue nitrile gloves during sampling. For every batch of experiments, three replicate clean Petri dishes and filter papers were placed on the bench close to the area of work to monitor any possible air contamination when taking out and thawing the intestine samples for FTIR analysis. In addition to these “air blanks”, procedure blanks were processed to monitor the potential contamination of reagents and/or glassware. All the sieves were rinsed with filtered Milli-Q water into a clean glass container as its corresponding sample. Then, it was processed synchronously with cetacean samples in subsequent experiments. If MPs found within the procedural blanks or air blanks matched the particle shape, color, and composition of any particle found within the corresponding sample, these particles would then be removed from final counts to correct the data.

### 2.4. Data Analysis

The abundance of MPs for each individual was reported by the number of plastic particles per individual and per gram of intestine contents (wet weight, ww). MPs composition, color, shape, and size were determined. Spearman rank correlation tests were performed to examine possible relationships between total MPs abundance and cetacean body length and relationships between total MPs abundance and intestinal content mass. Mann–Whitney U tests were used to determine whether the abundance of MPs differed according to the sex of the cetacean, and the ecological groups (deep-diving species vs. coastal species, but not including pelagic species, for which the sample size is not up to 3). Statistical analyses were performed using SPSS 25 software (IBM Corp., Armonk, NY, USA).

## 3. Results

### 3.1. Abundance of MPs

**Figure 2:**
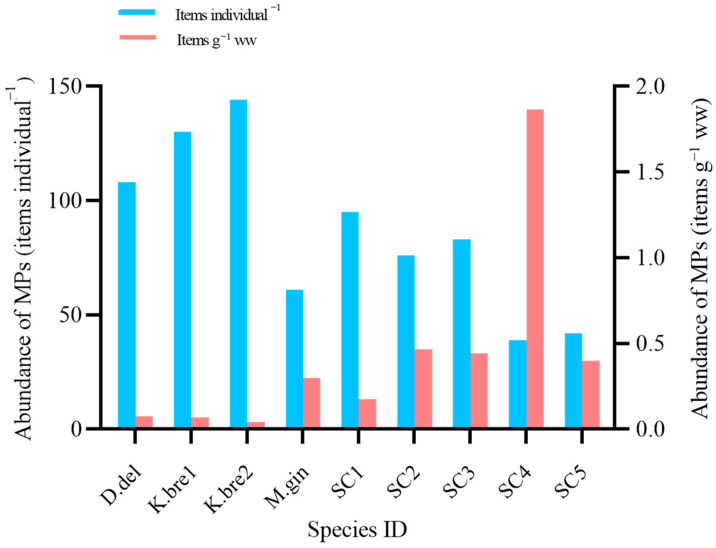
The abundance distribution of MPs in each cetacean. The blue columns are the abundances of MPs calculated per individual in each cetacean, and the red columns are the abundances of MPs calculated per unit wet weight of intestine contents in each cetacean.

**Figure 3:**
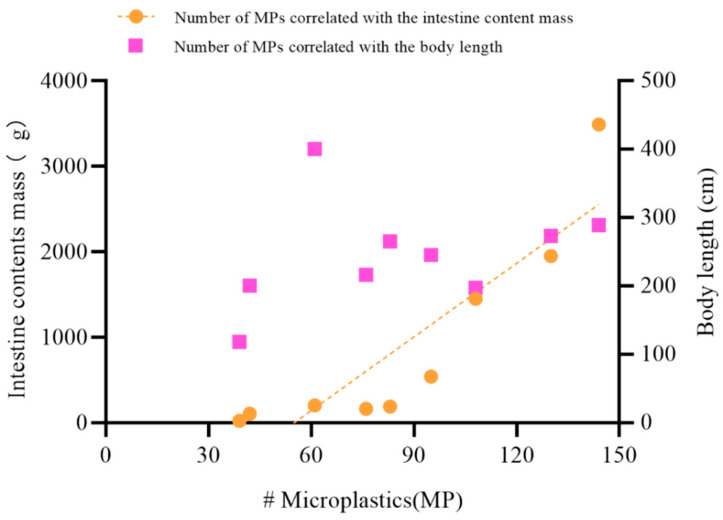
The correlation among MPs abundance, cetacean body size, and intestine contents mass. The pink squares represent the number of MPs correlated with the body length of each cetacean; the orange circles represent the number of MPs correlated with the intestine contents mass of each cetacean. The correlation curve is labeled with the dashed line (Spearman’s r = 0.95, *p* = 0.0004).

Cetacean recovery site, time, and biological parameters (body length, gender, intestinal length, mass of intestinal contents) and MPs abundance are detailed in Table 1. Due to the large number of intestine samples, we finished all the MPs isolation and identification work with five batches of experiments. We did not find contamination in any procedure blank. However, for the total 15 air contamination monitoring filters, we found two, one, and one MP item in 3 filters from two batch experiments. Therefore, MPs of the same shape and polymer type to those found in the samples were subtracted. Of 1425 suspected MPs particles sorted visually from all intestinal samples examined by Micro-FTIR individually, 55.1% were confirmed to be MPs. Finally, MPs were found in all nine specimens, with a total of 778 MPs items identified in all the intestines excluding the MPs for the blank (0.27 ± 0.60 items filter^−1^). The mean abundances were 86.44 ± 12.22 items individual^−1^ (i.e., 0.426 ± 0.188 items g^−1^ ww) for all samples (n = 9), ranging from 39 to 144 items individual^−1^ (i.e., 0.041 to 1.866 items g^−1^ ww). Though the highest abundance of MPs items were found in one pygmy sperm whale (K.bre2) calculated by individual (144 items individual^−1^), it was at the lowest level (0.041 items g^−1^ ww) calculated by intestine contents mass (Figure 2). There was a significant positive correlation between the number of MPs and intestine contents mass (Spearman’s r = 0.95, *p* = 0.0004 *), but there was no correlation between MPs abundance and cetacean body length (*p* = 0.23) (Figure 3). There were no apparent differences in MP counts between male and female specimens (Mann–Whitney U test, *p* = 0.064) or between deep-diving species and coastal species (Mann–Whitney U test, *p* = 0.071).

### 3.2. Characteristics of MPs

**Figure 4:**
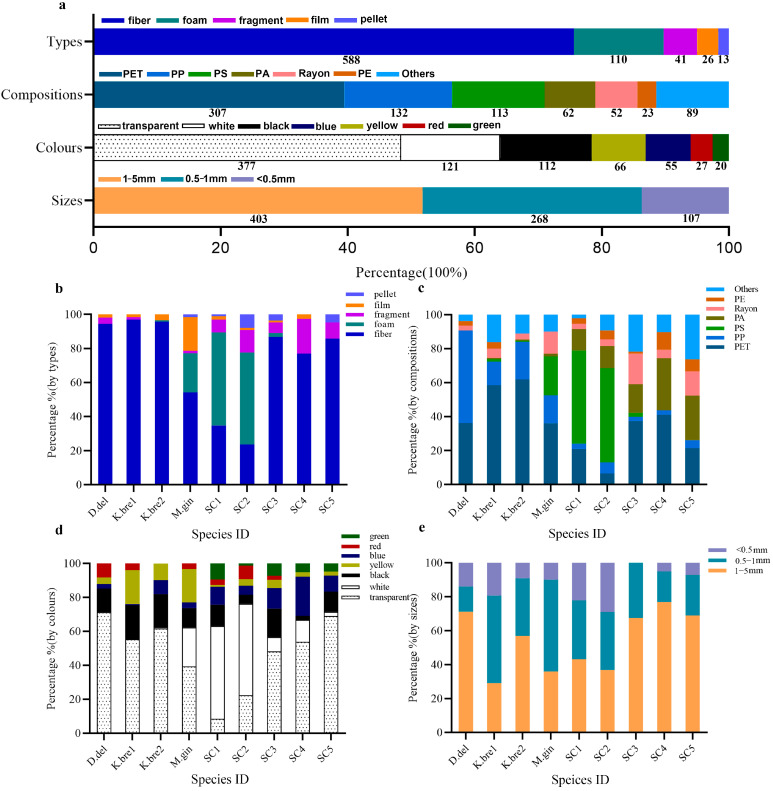
The percentages of MPs in total (**a**) and a comparison of MPs by shape (**b**), composition (**c**), color (**d**), and size (**e**) in different cetaceans. The numbers in (**a**) are the actual numbers of MPs belonging to different categorizations.

MPs were analyzed based on shape, color, polymer type, and size (Figure 4a). Five MPs shapes were present: fiber, film, foam, fragment, and pellet (Figure 4b). Of these, fibers were most common, accounting for an average of >75% of the total abundance of MPs, ranging 23.7–96.9% of MPs among individuals. The foam items were the second most. The foam-shaped MPs were found in those polystyrene (PS)-predominant individuals (M.gin, SC1, SC2) (Figure 4c), which is because the majority of PS foams were found in them. 

Most identified polymer types (Figure 4c) were polyethylene terephthalate (PET, 39.5%), polypropylene (PP, 17%), polystyrene (PS, 14.5%), polyamide (PA, 8%), rayon (6.7%), and polyethylene (PE, 3%) and comprised 88.6% of all MP types. Of them, PET, PP, and rayon occurred in each specimen. Though PS was a predominant MP type, PS foams occurred only in some individuals.

Different colored MPs (Figure 4d) included transparent (48.6%), white (15.6%), black (14.4%), yellow (8.5%), blue (7.1%), red (3.5%), and green (2.6%). Transparent MPs were prevalent and found in each individual, ranging 8.4–71.3%, except for in two humpback dolphins in which white items (>50% PS foams) were predominant. 

MPs occurred in all sieve fractions (Figure 4e), with 13.8% of items being smaller than 0.5 mm, 34.4% between 0.5 mm and 1 mm, and 51.8% between 1 mm and 5 mm. MPs between 0.5 mm and 5 mm comprised 86.2% of all MPs.

## 4. Discussion

Monitoring uptake of MPs by marine organisms is necessary to evaluate the risk they pose in the marine environment [46]. While MPs have been identified from filter-feeding baleen whales and teuthophagous and piscivorous toothed whales, these cetaceans can also be classified into the ecological groups of coastal, pelagic, and deep-diving species (Table 2) [10]. Using this latter classification, we reported MPs from the intestinal contents of pelagic and deep-diving cetaceans from Chinese waters for the first time.

Different feeding habits and different statistical analytical methods made it difficult to compare exposing risk levels of MPs from all different species. We tried to find published MPs data (usually 0.1–5 mm) from the same cetacean species or species with similar habits from waters worldwide (Table 2) and made a rough comparison of the MPs abundance (MPs usually calculated by individual in the published data). For the coastal species Indo-Pacific humpback dolphins, we showed that the abundance of MPs (67 ± 25 items individual^−1^) from Xiamen Bay in this study was a bit higher than previously reported data for this species from the Pearl River Estuary (53 ± 35.2 items individual^−1^) [25] and Beibu Gulf (37.5 ± 7.5 items individual^−1^) [26] and also higher than in finless porpoises (19.1 ± 7.2 items individual^−1^) from Yellow Sea and Bohai Sea, China [24], and much higher than harbor porpoises (5.24 ± 2.53 items individual^−1^) from the British coast [47]. The abundance of 108 items individual^−1^ that we reported for the common dolphin in this study far exceeded the MPs abundance reported for this species from other waters, including the Galician coast (3–41 items individual^−1^) [22], the British coast (3 -12 items individual^−1^) [47], and New Zealand waters (1–21 items individual^−1^) [30]. For two pygmy sperm whales, the abundance of 137 ± 10 items individual^−1^ was also higher than this species from the British coast (4 items individual^−1^) [47] and the eastern North Atlantic (59.08 ± 40.52 items individual^−1^) [28]. According to the previous studies, the western Pacific Ocean presenting a high concentration of plastic debris [2] might contribute the high MPs abundance in marine species in this area. However, it should be noted that beyond the physical conditions of the animals (e.g., age, body size, and the cause of death), the different MP extraction methods (such as target MPs size range), sampling organs (entire GIT or only a section), and suspected MPs verification percents (if 100% suspected MPs have been confirmed) may also affect MPs abundance.

Some previous studies tried to examine the possible relationships of MPs abundance with length or size of the dolphins. While no correlation has been reported between dolphin size and the number of MPs within stomachs [22,40], but a positive correlation has been reported between MPs abundance and the mass of contents within intestinal samples of stranded bottlenose dolphins [48]. In this study, we also found that there was a strong significant relationship between MPs abundance and intestine contents mass (*p* < 0.01) in the nine cetaceans (Figure 3). In addition, the abundance level of MPs in cetacean samples presented by individuals was probably different from calculated by intestine contents mass (Figure 2). Therefore, we suggest that intestine contents mass should be reported in addition to cetacean size in future MPs studies. MPs databases for stranded cetaceans with more detailed physical information will enable a more comprehensive and effective spatio-temporal MPs pollution comparison for cetaceans, rather than only comparing MPs abundance by individuals. In addition, different intestinal parts with special structure might impact the MPs accumulation [24]. However, we did not analyze the MPs in the different intestinal parts due to the limited intestinal structure information of these species in this study. We will pay more attention to the number of MPs per intestinal part in the future studies.

Determining the exact origin of the MPs was relatively difficult because of the complexity of their migration, transport, and transformation in the marine environment [49]. However, polymer type, shape and color, and the spatial distribution of MPs can be used to infer MPs origin [50,51]. Fibers were the most (more than 75% of all MPs items) abundant shape in cetacean intestine contents, consistent with previous accounts of MPs in marine mammals [15,22,26]. MPs have been reported from both cetacean gastrointestinal tracts and their feces [10,15,23], indicating that a few MPs were passing through the GIT and egested [47]. And the retention of fibrous MPs might be related to the wrinkled structure of the intestinal walls of the cetacean, wherein fibers might become more easily trapped [24]. Fibers were reported as the main type of MPs pollution in waters and sediments [52]. The widespread occurrence of fibrous MPs in the marine environment may explain their dominance in both cetaceans [44,47] and fishes [45,53]. As fibers are widely used primary raw materials in the textile industry, many fibrous MPs came from textile laundry wastewater [51,54]. In addition, both fishing nets and ropes were further possible sources [55]. 

Most (79%) of the polymers that we identified were composed of polyethylene terephthalate (PET, 39.5%), polypropylene (PP, 17%), polystyrene (PS, 14.5%), and polyamide (PA, 8%). Differences in MPs polymer type may be related to the survey area [23]. The prevalence of PET and PP accounting for more than 55% in this study was consistent with the main polymer types recently reported in seawater from this region [44,56]. As teuthophagous and piscivorous predators, the toothed whales mainly ingest MPs through trophic transfer from prey [15]. The polymer PET was also usually found dominated in fishes from different Chinese waters, such as two major cities in Fujian Province from the western coast of the Taiwan Strait [13], Hangzhou Bay and the Yangtze Estuary [57], and the Pearl River Estuary [58]. As a form of polyester, more than 80% PET from 42 × 10^6^ t of synthetic fibers has been estimated to be produced in textiles annually [38,59,60]. Therefore, the widespread distribution of PET fibers might be because Fujian province along the western coast of the Taiwan Strait had a large textile industry, where shoe and garment industries caused the discharge of PET-rich wastewaters into the marine environment [37]. PP was the second most common polymer found in our study, and it also has been reported for other cetacean species in China, such as finless porpoises in the Yellow and Bohai Seas [24], and humpback dolphins in the Pearl River Estuary [25]. PP was widely used in industrial, residential, and fishing activities, such as for some automotive parts, in textiles and for plastic bags, and for fishing ropes and nets [61]. The PP MPs in this study may also have originated from either textile industries or fishery activities in and around the Taiwan Strait. 

The colors of MPs varied by region and species. Consistent with many previous studies, transparent, white, and black colors were commonly found in this study [45,62], in which transparent items were dominated (48.58%). The commonality of clear plastics in fishing lines and nets, packaging, clothing [63,64], and the whitening and yellowing effect caused by environmental physicochemical processes, including aging and photolysis [25] and discoloration by alkalinity [48,63], may increase the proportion of colorless items.

In the present study, MPs occurred in all sieve fractions, and most MPs were identified in the size ranges of 1–5 mm (51.8%) and 0.5–1 mm (34.4%), similar to those in cetaceans reported in previous studies [21,22,25,26]. Usually, plastic fragments in the marine environment are gradually broken into smaller particles by wave action, photo-oxidation, and biodegradation [65]. The higher percentage of large size MPs in humpback dolphins in this study might be due to the relatively short transport distance for plastic wastes from the city sewage treatment plants in Xiamen Bay [25]. Moreover, mounts of large-size fibers were also found in the other pelagic and deep-diving cetaceans in this study. Because we have adapted washing protocols using different size range nested sieves to collect intestine contents previously used for many other cetacean taxa [10,15,22,24,44,48], the smallest size of MPs found was 0.102 mm, just above the smallest sieve size. Similar to our results, the smallest size of MPs reported in many published papers about cetaceans was usually above 0.1mm [15,26,28,47]. However, there were still some smaller MPs items that might pass through the smallest size mesh. In future studies, we will focus more on those smaller items. Although MPs would not cause physical obstacles to cetaceans through entanglement or swallowing like large plastic fragments, they could embed within tissues and induce local gastrointestinal tract damage [23]. Additionally, MPs remaining in the intestines of cetaceans could also act as carriers of toxic substances, such as heavy metals and persistent organic pollutants, which were bioaccumulated in top predators [66,67] and could be deleterious to health [68,69].

In this study, we have taken extensive measures to minimize the risk of contamination of samples by airborne MPs or from use of equipment. We have also used “air blanks” to monitor the air contamination and procedure blanks to monitor potential contamination of reagents and/or glassware during the gut content extraction, MPs isolation, and analysis process. However, there were still potential contamination risks while dissecting the cetacean carcasses and taking out the intestines. Otherwise, the aluminum foil wrapping the intestines and the sealed bags might be other potential contamination sources. Although we have rinsed the intestines from the outside with filtered Milli-Q water before opening them, the contamination risks from the postmortem examination and the intestine collection step cannot be ruled out. In future studies, we should rinse or pre-burn the aluminum foil to avoid contamination and place blanks during the whole process from the postmortem examination to finish analyzing MPs with FTIR.

## 5. Conclusions

MPs marine pollution is ubiquitous, and MPs are now commonly identified in apex marine predators such as cetaceans. Monitoring MPs in cetaceans is challenging because of difficulties in obtaining samples, especially for endangered species. Therefore, we investigated MPs in nine individuals of four cetacean species stranded along the western Taiwan Strait. It provided the first report of MPs from pelagic and deep-diving cetaceans in China and the first report anywhere of MPs from ginkgo-toothed beaked whale. Transparent, fibrous MPs made from PET and PP were found to be predominant in this study. Synthetic fibers might be the primary MPs source, discharged into the marine environment and transferred to cetaceans via municipal and industrial sewage from clothing production and industry along the western coast of the Taiwan Strait and from fisheries and aquaculture. The presence of MPs in ecologically important indicator species such as cetaceans highlights the need to better understand their sources and impacts throughout food chains and harmful effects on apex predators.

## Figures and Tables

**Table 1:** Stranding location, time, biological parameters, and MPs abundances of the cetaceans.

Id	Species	Stranding Location	Stranding Date	Gender	Body Length (cm)	Collected Contents Mass (g)	Collected Intestinal Length (m)	MPs Items/ Individual	MPs Items/Wet Weight (g)	Decomposition State
D.del	*Delphinus delphis*	119.89° E 26.38° N	7 Jan 2019	Male	197.3	1449.0	19.5	108	0.075	II
K.bre1	*Kogia breviceps*	119.67° E 25.62° N	27 Jun 2019	Female	273.2	1948.4	20.3	130	0.067	II
K.bre2	*Kogia breviceps*	119.76° E 25.36° N	3 Apr 2019	Female	289.0	3489.4	28.4	144	0.041	III
M.gin	*Mesoplodon ginkgodens*	119.89° E 25.51° N	9 Jul 2019	Female	400.0	203.2	2.4	61	0.300	II
SC1	*Sousa Chinesis*	118.45° E 24.61° N	15 Mar 2016	Female	245.0	538.4	9.3	95	0.176	II
SC2	*Sousa Chinesis*	118.16° E 24.60° N	5 Mar 2019	Male	216.0	162.4	7.9	76	0.468	II
SC3	*Sousa Chinesis*	118.05° E 24.47° N	15 Apr 2020	Male	265.0	188.6	15.5	83	0.440	II
SC4	*Sousa Chinesis*	118.21° E 24.47° N	13 Jul 2021	Male	118.0	20.9	7.3	39	1.866	III
SC5	*Sousa Chinesis*	118.15° E 24.42° N	8 Oct 2021	Male	200.0	105.8	12.6	42	0.397	III

MPs items/individual: The abundance of MPs was reported by the number of plastic particles per individual; MPs items/wet weight (g): The abundance of MPs was reported by per gram of intestine contents (wet weight, ww).

**Table 2:** Comparison of the MPs abundance in cetaceans from different regions around the world.

Species	Organ	Sample Number	Body Length (cm)	MPs Items /Individual	Range MPs/Animal	Size Range (mm)	Confirmation Method (% Analyzed)	Habitat	Location (References)
Indo-Pacific humpback dolphin (*Sousa chinensis*)	intestine	5	209 ± 57	67 ± 25	39–95	0.1–5	FTIR (100%)	Coastal	Western coast of the Taiwan Strait (this study)
Indo-Pacific humpback dolphin (*Sousa chinensis*)	stomach	12	241.2	53 ± 35.2	11–145	<5	FTIR (100%)	Coastal	Pearl River Estuary, China ([25])
Indo-Pacific humpback dolphin (*Sousa chinensis*)	intestine ^a^	3	213.5	37.5 ± 7.5	2–45	0.1–4.8	FTIR (100%)	Coastal	Beibu Gulf, China ([26])
East Asian finless porpoise (*Neophocaena asiaeorientalis sunameri*)	intestine	7	143 ± 40	19.1 ± 7.2	10–32	0.125–5	Raman (100%)	Coastal	Yellow Sea and Bohai Sea, China ([24])
Harbor porpoise (*Phocoena phocoena*)	GIT	21	128 ± 18	5.24 ± 2.53	2–11	0.1–5	FTIR (not 100%)	Coastal	British coast ([47])
Common dolphin (*Delphinus delphi*s)	intestine	1	197.3	108	108	0.1–5	FTIR (100%)	Pelagic	Western coast of the Taiwan Strait (this study)
Common dolphin (*Delphinus delphis*)	stomach	35	None reported	12 ± 8	3–41	0.29–5	None reported	Pelagic	Galician coast ([22])
Common dolphin (*Delphinus delphis*)	GIT	16	191 ± 21	5.69 ± 3.34	1–12	0.1–5	FTIR (not 100%)	Pelagic	British coast ([47])
Common dolphin (*Delphinus delphis*)	stomach	15	184 ± 29	7.8 ± 1.4	1–21	0.04–10	FTIR (100%)	Pelagic	New Zealand waters ([30])
Striped dolphin (*Stenella coeruleoalba*)	intestine	43	160.31 ± 66.38	14.9 ± 22.3	1–82	0.1–5	FTIR (not 100%)	Pelagic	Western Mediterranean Sea ([34])
Pygmy sperm whale (*Kogia breviceps*)	intestine	2	281 ± 11	137 ± 10	130–144	0.1–5	FTIR (100%)	Deep-diving	Western coast of the Taiwan Strait (this study)
Pygmy sperm whale (*Kogia breviceps*)	entire GIT	1	164	59.08 ± 40.52 *	None reported	0.2–5	Raman (not 100%)	Deep-diving	Eastern North Atlantic ([28])
Pygmy sperm whale (*Kogia breviceps*)	GIT	1	211	4	4	0.1–5	FTIR (not 100%)	Deep-diving	British coast ([47])
True’s beaked whale (*Mesoplodon mirusa*)	GIT	3	500	88	88	0.3–5	FTIR (not 100%)	Deep-diving	Ireland coast ([15])

GIT: stomach and intestines; entire GIT: from oesophagus to anus; ^a^: MPs abundance analyzed by a subsample; * average MPs abundance within study including multiple species.

## Data Availability

The data that support the findings of this study are available within the article. Any further information is available from the corresponding author upon reasonable request.
